# Usefulness of bone scintigraphy for the diagnosis of Complex Regional Pain Syndrome 1: A systematic review and Bayesian meta-analysis

**DOI:** 10.1371/journal.pone.0173688

**Published:** 2017-03-16

**Authors:** Maria M. Wertli, Florian Brunner, Johann Steurer, Ulrike Held

**Affiliations:** 1 Horten Centre for Patient Oriented Research and Knowledge Transfer, University of Zurich, Pestalozzistrasse 24, Zurich, Switzerland; 2 Division of General Internal Medicine, Bern University Hospital, Bern University, Freiburgstrasse 8, Bern, Switzerland; 3 Department of Physical Medicine and Rheumatology, Balgrist University Hospital, Forchstrasse 340, Zurich, Switzerland; BG Trauma Center Ludwigshafen, GERMANY

## Abstract

**Background:**

Since 2007, the Budapest criteria are recommended for the diagnosis of Complex Regional Pain Syndrome (CRPS) 1. The usefulness of bone scintigraphy (BS, index test) for the diagnosis of CRPS 1 remains controversial. Imperfect reference tests (RT) result in underestimation of the diagnostic accuracy of BS. Further, biased results can occur when a dependency between the RT and BS exists.

The objective was to assess the impact of different RTs, specifically the Budapest criteria, and the assumed imperfect nature of the RT on the diagnostic accuracy of BS. Further, we analyzed the association between baseline characteristics and positive BS in patients with CRPS 1.

**Methods:**

Systematic literature review and Bayesian meta-analysis to assess the test accuracy of BS with and without accounting for the imperfect nature of the RT. We examined correlations (Spearman correlation coefficients / Wilcoxon tests) between baseline characteristics and the proportion of positive BS in patients with CRPS 1.

**Results:**

The pooled sensitivity was 0.804 (95% credible interval (CI) 0.225–1.0, 21 studies) and specificity 0.853 (95%CI 0.278–1.00). Sensitivity and specificity of BS increased when accounting for the imperfect nature of the RT. However, in studies using Budapest criteria as reference, the sensitivity decreased (0.551; 95% CI 0.046–1) and the specificity increased (0.935; 95% CI 0.306–1). Shorter disease duration and a higher proportion of males were associated with a higher proportion of positive BS (27 studies, disease duration <52 weeks Wilcoxon test p = 0.047, female proportion Spearman correlation −0.63, p = 0.009).

**Conclusion:**

Compared to the accepted Budapest diagnostic criteria BS cannot be used to rule-in the diagnosis of CRPS 1. In patients with negative BS CRPS 1 is less likely the underlying illness. Studies using older or no diagnostic criteria should not be used to evaluate the diagnostic accuracy of BS in CRPS 1.

## Introduction

Complex Regional Pain Syndrome (CRPS) is a painful disorder characterized by sensory, autonomic, motor and trophic changes.[1] Two types of CRPS are defined by absence (CRPS 1) or presence of a definable nerve lesion (CRPS 2). Since 2007, the Budapest criteria, clinical criteria based on signs and symptoms, are recommended for the diagnosis of CRPS 1.[2] Although the prevalence of CRPS 1 is low, the patient burden in those suffering from the disease is high and associated with substantial direct medical and social costs (e.g. loss of productivity, disability, pension payments).[1, 3, 4] Despite the overall good response to treatment, one third of the CRPS 1 patients will not improve and develop chronic disease with substantial pain, disability, and impaired quality of life.[3] Late diagnosis and incorrect treatment contribute to the development of chronic CRPS 1 while early treatment is associated with better course.[4] Therefore, an early diagnosis of CRPS 1 is of great importance.

Despite a broad consensus that CRPS 1 is a clinical diagnosis based on the Budapest criteria [2], some authors recommend the use of bone scintigraphy (BS) to confirm the CRPS 1 diagnosis.[4–6]The usefulness of BS as diagnostic test in CRPS 1 remains controversial. While some studies found a high sensitivity of a positive BS (increased periarticular uptake) [6–8] a recent Meta-analysis concluded that BS does not add any benefit to the clinical diagnosis of CRPS 1 and should not be used for confirmatory purposes.[9] Due to the broad spectrum of clinical manifestations the diagnosis of CRPS 1 remains a challenge in daily clinical practice and a single test to confirm or to rule out the disease would be most helpful.

In diagnostic Meta-analyses results of diagnostic studies are pooled to improve the estimates accuracy by using as many available studies as possible. However, various aspects may reduce the confidence in the pooled estimate. The test performance of BS is underestimated when Meta-analyses fail to account for the imperfect nature of a reference tests (i.e. clinical criteria for the diagnosis of CRPS 1.[10] Overestimation of the test performance occur in studies with a high disease prevalence [11] or when a dependency between the reference test and index test exists.[12, 13] To date, no study has assessed the impact of the different diagnostic reference standard tests used for the diagnosis of CRPS 1 on the diagnostic accuracy of BS. Further, it is unclear whether patient characteristics influence the proportion of positive BS results. Previous meta-analyses did not account for the prevalence of the diseases, the imperfect nature of the reference standard and other covariates that may influence the test accuracy.[10] Bayesian meta-analytical methods offer the advantage to account for various factors including the disease prevalence, the imperfect nature of the reference standard, and covariates.

Therefore, the objective of this study was to demonstrate the impact of the different reference standard tests (diagnostic criteria) on the pooled sensitivity and specificity of BS for the diagnosis of CRPS 1 using novel Bayesian meta-analytical methods that account for the prevalence of the disease and the imperfect nature of the reference standard test. Further, we assessed the association between patient characteristics and the proportion of positive bone scans.

## Methods

The systematic review and meta-analysis of diagnostic studies was conducted in accordance with the recommendations by the Preferred Reporting Items for Systematic Reviews and Meta-Analyses (PRISMA statement, S1 Table.).[14, 15]

### Literature search

We identified diagnostic studies in patients with CRPS 1, published between the inception and July 2015, by searching the following databases: MEDLINE (OvidSP), MEDLINE In-Process Citations (OvidSP), Embase (Elsevier), Cochrane Database of Systematic Reviews (Wiley), Cochrane Central Register of Controlled Trials (CENTRAL), CINAHL (EBSCO), Scopus (Elsevier). The terms for the search strategies were identified through discussion between an information specialist and the review team, by scanning the background literature, and by browsing the MEDLINE Thesaurus (MeSH). Three detailed search strategies are described in S2 Table. To ensure the completeness of the literature search, the reviewers, experienced clinicians and researchers in the field of CRPS 1, screened bibliographies of all included studies, retrieved review articles and current treatment guidelines in an additional hand search and all potentially eligible references were included in the full text review (inclusion and exclusion criteria applied).

### Eligibility criteria

Eligible were studies that investigated the utility of bone scintigraphy for the diagnosis of CRPS 1. To investigate the diagnostic accuracy of bone scintigraphy all studies that reported sensitivity and specificity or the numbers needed to calculate sensitivity or specificity were included, regardless of reference standard. Excluded were studies where data on sensitivity and specificity could not be extracted.

To analyze patient characteristics associated with a positive bone scan, studies using IASP diagnostic criteria or more recent ones (see description below) were eligible. We included studies that reported sensitivity and specificity. In addition we also included studies on patients with established diagnosis of CRPS 1 (in which only sensitivity of BS could be assessed).

### Study selection

Two reviewers (MW and FB) independently screened 725 references by title and abstract to identify studies to be included according to the inclusion criteria. Disagreements were discussed and resolved by consensus of the authors or third party arbitration (UH). All full texts of studies potentially meeting the inclusion criteria or where inclusion was unclear were then obtained and reviewed in full text by the two reviewers (MW and FB) independently. Again, disagreements were discussed and resolved by consensus or by third party arbitration (UH). In the case of several publications for the same patient population the most recent publication was chosen and missing information from the previous publications added. No language restriction was set. Alternative researchers with specific language proficiencies were used for non-English language references.

### Data extraction and synthesis

We extracted the following variables from each study: author, publication year, country of origin of the study, study population demographics, reference standard (presence or absence of CRPS 1 based on clinical criteria), index test (positive or negative BS), the corresponding absolute numbers of true-positive (TP), false-positive (FP), false-negative (FN), true-negative (TN), and total number of patients.

### Methodological quality and risk of bias

The quality of the diagnostic studies was assessed by using the SIGN quality check list [16] that is in accordance with the recommendations by the revised tool for the Quality Assessment of Diagnostic Accuracy Studies (QUADAS-2).[13] Two reviewers (MW, FB) independently assessed the methodological quality of each study. We did not exclude studies based on their quality rating. In particular we did not exclude studies without clearly defined reference standard test because this was part of the research question addressed in this study.

The overall methodological quality of the study was rated as follows: High quality (++): Majority of criteria met (little or no risk of bias, results are unlikely to be changed by further research); Acceptable quality (+): Most criteria met. Some flaws in the study with an associated risk of bias, Conclusions may change in the light of further studies; Low quality (-): Either most criteria not met, or significant flaws relating to key aspects of study design. Conclusions likely to change in the light of further studies. Studies that did not meet the predefined criteria in six or more out of 13 domains were rated as low quality.

### Diagnostic test under investigation

Radionuclide bone scintigraphy (BS) is a relatively inexpensive, widely available, and valuable procedure in the diagnostic evaluation of numerous illnesses.[17] BS is performed by injecting technetium-99m–labeled diphosphonates intravenously. The administered activity for adult patients is between 740 and 1,110 MBq (20–30 mCi).[18] Imaging is conducted at three time points: Flow images (during injection), blood pool images (3–5 minutes after injection), and the delayed (skeletal phase) images 2–5 hours after injection.[18] Interpretation criteria include: increased or decreased tracer activity in the bone, change in focal abnormalities to previous studies, and soft tissue (e.g. generalized interstitial uptake compared with normal bone or focal tracer uptake in organs).[18]

### Reference tests

In the past, several diagnostic criteria have been introduced for the diagnosis of CRPS 1. A summary of most prevalent criteria [2, 19–23] is provided in S3 Table. In 1994 the International Association for the study of pain (IASP) introduced the most recent definition of CRPS together with an expert agreed set of diagnostic criteria. The IASP criteria showed a high sensitivity (1.0) with a low specificity (0.41) that bared the risk of an over-diagnosis.[19, 24] Bruehl et al. proposed modified diagnostic criteria in 1999.[19] In 2003 the IASP were updated and published as Budapest criteria in 2007 [2] and further adapted for research by Harden et al. in 2010.[24] The Budapest criteria showed a high sensitivity (0.99) and an improved specificity (0.68)[24] and are recommended for the diagnosis of CRPS 1 by current guidelines.[25, 26]

### Statistical analysis

The complexity of the data requires the use of random effects models. Based on the imperfect nature of the diagnostic criteria as reference test we used two models to analyze the diagnostic accuracy: One without and one accounting for the imperfect nature of the reference tests. We used a hierarchical Bayesian model, as proposed by Dendukuri et al. [27], which accounts for the within study and between-study variability and the potentially imperfect nature of the different reference tests. The models were compared using summary ROC curves. The hierarchical Bayesian model was set up as follows: we assumed *j* = 1,…*J* diagnostic studies in the meta-analysis, with cross tabulation between index test (T1, here bone scintigraphy) and reference test (T2) available for each study, and both tests assumed to be dichotomous (1 = positive test result, 0 = negative test result). Each study was assumed to use a different cut-off value (*θ*_*j*_) to define a positive test result. The diagnostic accuracy of each study was denoted by *α*_*j*_. The model structure implied a within-study level for study-specific parameters (*θ*_*j*_ and *α*_*j*_), and a between-study level for global parameters common among all studies. The estimated study-specific parameters for accuracy and threshold, together with global parameters could be used to recalculate sensitivity and specificity of the index test in study *j*. Details of the model formulations can be found in the publication by Held et al.[10]

Results of the Bayesian analysis are samples from the posterior distribution of the unknown parameters–especially sensitivity and specificity, and estimates are presented as posterior medians (50% quantile), and lower (2.5% quantile) and upper (97.5% quantile) bounds, resulting in a 95% credible region. The width of the credible region is an indicator for heterogeneity of the studies.

To assess the patient characteristics that influence the likelihood for a positive scintigraphy we jointly analyzed data from studies reporting sensitivity and specificity of BS and data from studies that reported sensitivity alone (13 studies). The following factors were defined a priori: mean duration of symptoms (continuous and dichotomous for <52 weeks / ≥52 weeks), mean age (continuous), study design (prospective, retrospective), gender, and location (upper extremity vs. mixed location). We used Spearman correlation coefficients for continuous variables and Wilcoxon tests for dichotomous variables to determine whether sensitivity of BS was associated with any of the above factors.

All analyses were performed with the statistical software R and the package HSROC.[28]

### Ethical review board approval

For this study no ethical approval was required. No protocol was published or registered. All methods were determined a priori.

## Results

### Study selection

**Fig 1:**
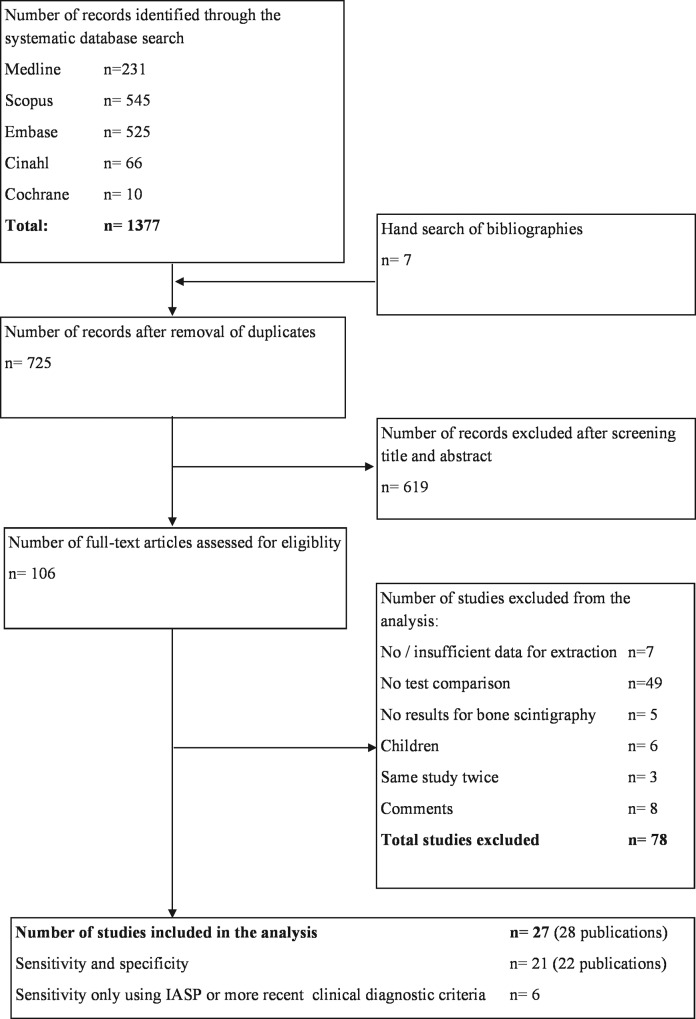
Study Flow.

The systematic search retrieved 725 potentially eligible studies. After screening title and abstract, 106 articles were read in full text by rigorously applying the inclusion and exclusion criteria (**Study Flow Fig 1**). The main reasons for exclusion are summarized in Fig 1 and included no reference standard test or test comparison (n = 49) and no extractable table (n = 7). Finally, 21 diagnostic studies (22 publications) and 6 studies reporting bone scintigraphy results in patients with CRPS 1 met our criteria and were included in our analysis.

### Study characteristics

In studies on diagnostic accuracy of BS the study design was prospective in seven studies [29–34] retrospective in 13 studies, [35–45] and mixed prospective and retrospective in one study.[46] The sample size ranged from 13 to 145 patients (Table 1), mean age from 35 to 63 years, and the average disease duration from 6 to 103 weeks. The reference standard for the diagnosis of CRPS was in three studies the clinical Budapest criteria, [29, 44, 45] four studies the IASP criteria, [34, 42, 43, 47] and in five studies the Kozin diagnostic criteria.[40, 41, 46, 48, 49] Seven studies [30–32, 36–38] did not report on the diagnostic criteria used for the diagnosis of CRPS 1 and two studies [33, 39] used other clinical criteria In total 13 studies were included in the analysis of the influence of patient characteristics on the proportion of positive bone scans. [29, 34, 42, 43, 45, 47, 50–56] We included five studies using the IASP criteria [34, 52–55] and one study using the Budapest criteria [51] that reported results on the sensitivity of BS in patients meeting the clinical diagnostic criteria for the diagnosis of CRPS 1. The extracted information for each study used for the diagnostic meta-analysis is provided in S4 Table.

**Table 1:** Baseline characteristics of patients included in the studies

**Studies reporting on sensitivity and specificity**										
**ID**	**Author, year**	**n (female)**	**Age (mean)**	**Duration Symptoms (weeks)**	**Initiating event**	**Localization**	**Reference Test**	**Index Test**	**Dose**	**Design**
2	Kozin, 1981 [30]	50 (28)	48.3	75.9	Miscellaneous	UE, LE	NR	3-phase Tc-99m MDP	15 mCi	Prospective
3	Leitha, 1996 [63]	120 (82)	50.1	36.8	Miscellaneous	UE, LE	NR	5-phase Tc-99m MDP	600 MBq	Prospective
4	O'Donoghue, 1993 [32]	78 (NR)	NR	NR	NR	UE	NR	3-phase Tc-99m MDP	550–710 MBq	Prospective
8	Schiepers, 1998 [35]	50 (27)	44	NR	Trauma, surgery	UE	NR	3-phase Tc-99m MDP	740 MBq	Retrospective
10	Todorovic, 1995 [36]	44 (NR)	51	8.8	Fracture	UE, LE	NR	3-phase Tc-99m DPD	555 MBq	Retrospective
16	Weiss 1993 [37]	22 (NR)	NR	NR	CVI*	NR	NR	3-phase Tc-99m MDP	20mCi	Retrospective
19	Mackinnon 1983 [38]	145 (NR)	43	NR	Miscellaneous	UE	NR	3-phase Tc-99m MDP	20mCi	Retrospective
5	Okudan, 2005 [33]	34 (17)	61	6.5	CVI	UE	Clincial criteria	3-phase Tc-99m MDP	600 MBq	Prospective
11	Wang, 1998 [39]	30 (9)	63	6.1	CVI	UE	Clinical criteria	3-phase Tc-99m DPD	20 mCi	Retrospective
1 A+B	Tepperman 1984 and Greyson 1984 [48, 64]	85 (37)	60	9	CVI*	UE	Kozin	3-phase Tc-99m MDP	15 mCi	Retrospective
15	Werner 1988 [49]	63 (NR)	38	84	Miscellaneous	UE	Kozin	3-phase Tc-99m MDP	15mCi	Retrospective
17	Holder 1992 [46]	138 (18)	43.5	24	Miscellaneous	LE	Kozin	3-phase Tc-99m MDP	25mCi	Retro- and prospective
13	Constantinesco 1986 [65]	128 (61)	51	23	Miscellaneous	Hand	Kozin	3-phase Tc-99m MDP	200uCi/kg	Retrospective
18	Davidoff 1989 [41]	119 (65)	35.1	103.6	Miscellaneous	UE, LE	Kozin	3-phase Tc-99m MDP	15mCi	Retrospective
6	Park, 2007 [42]	38 (13)	52.2	0.3	CVI, TBI	UE	IASP	3-phase Tc-99m MDP	750 MBq	Retrospective
7	Park, 2009 [34]	50 (27)	56	13	CVI, TBI	UE	IASP	3-phase Tc-99m DPD	580–620 MBq	Prospective
9	Schurmann, 2007 [47]	107 (75)	59.9	16	Trauma	UE	IASP	3-phase Tc-99m MDP	580-620MBq	Prospective
21	Kim 2015 [43]	10 (5)	51	NR	NR	LE	IASP	3-phase Tc-99m MDP	20 mCi	Retrospective
12	Wüppenhorst, 2010 [29]	57 (38)	50.7	56.8	Trauma, surgery, Spontaneous	UE	Bruehl	3-phase Tc-99m MDP	500–700 mBq	Prospective
14	Moon 2012 [44]	116 (50)	40.5	53.6	Miscellaneous	UE, LE	Budapest	3-phase Tc-99m MDP	740MBq	Retrospective
20	Kwon 2011 [45]	140 (60)	39	64	Miscellaneous	UE, LE	Budapest	3-phase Tc-99m MDP	740 MBq	Retrospective
**Studies reporting on factors of influence for positive bone scintigraphy in patients with CRPS diagnosis**										
**ID**	**Author, year**	**n (female)**	**Age (mean)**	**Duration Symptoms (weeks)**	**Initiating event**	**Localization**	**Reference Test**	**Index Test**	**Dose**	**Design**
44	Konzelmann 2013 [51]	15 (5)	43	31	Non-traumatic	Hand	Budapest	3-phase Tc-99m MDP	NR	Retrospective
22	AlSharif 2012 [52]	37 (16)	38.8	25	NR	UE, hand	IASP	3-phase Tc-99m MDP	500–700 MBq	Retrospective
40	Handa 2006 [53]	14 (6)	55	12	Non-traumatic	UE (93%)	IASP	3-phase Tc-99m MDP	NR	Retrospective
53	Sampath 2013 [54]	68 (38)	43	57	Miscellaneous	UE, LE	IASP	3-phase Tc-99m MDP	740 MBq	Retrospective
55	Sezer 2008 [54]	24 (19)	52	5.6	Trauma	UE, LE	IASP	3-phase Tc-99m MDP	20 mCi	Prospective
26	Bruehl 2002 [56]	38 (24)	41	104	Trauma	UE, LE	IASP	3-phase Tc-99m MDP	NR	Retrospective

*thrombosis, embolism, hypertensive hemorrhage

UE, upper extremity; LE, lower extremity; NR, not reported; CVI, cerebrovascular insult; TBI, traumatic brain injury; MBq, millibecquerel (SI unit of measurement of radioactivity); mCI, millisievert (SI unit of measurement of readioactivity, 1 Bq = 0.027 × 10–9 CI).

### Study quality

One study met 12 of the 13 quality domains and was rated as high quality [29]. Six studies were rated low quality. Five studies [35–37, 39, 56] because they did not meet the quality in six or more domains and the risk of bias was substantial. Further, the study by Kim et al. [43] was downgraded from moderate to low quality because of the small sample size (10 patients with bone scintigraphy). Most studies (n = 20) were of moderate quality with some flaws associated with a risk of bias. The authors felt confident that in most studies the conclusion may change in the light of future studies (S5 Table

### Diagnostic accuracy of BS under the condition of a perfect and imperfect reference standard

**Fig 2:**
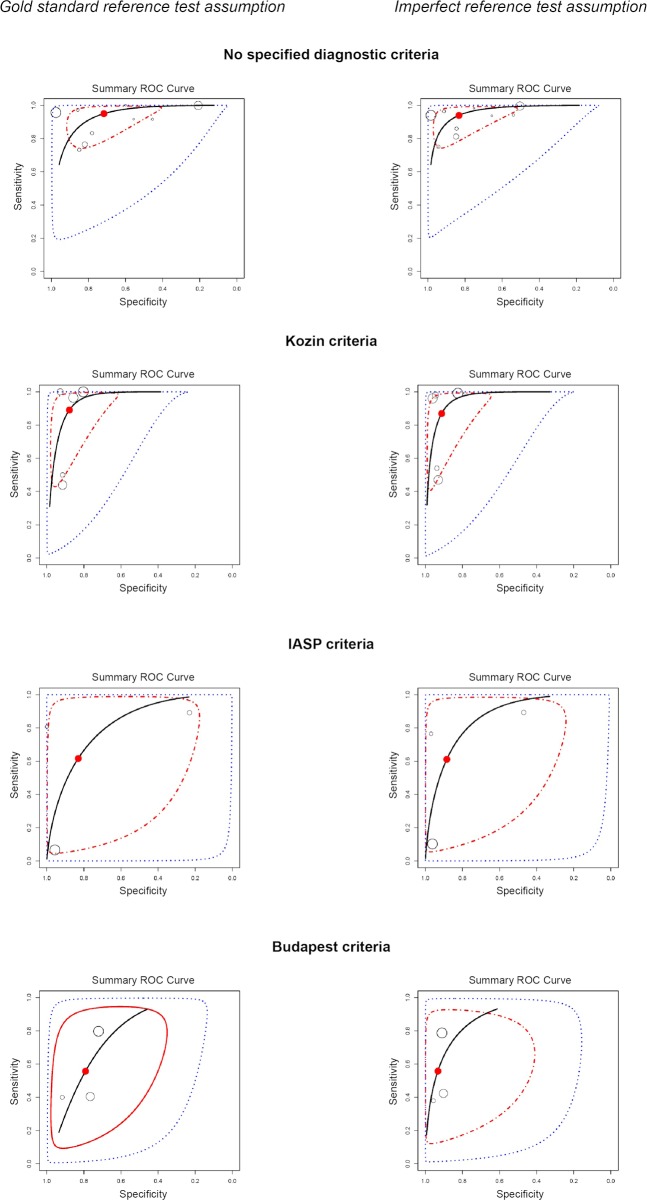
Summary receiver operating characteristic (ROC) curves for the joint meta-analysis of 21 studies. Results of the joint meta-analysis of 21 studies are presented by the overall posterior sensitivity and specificity with the corresponding 95% credible region (CI)

The joint meta-analysis of 21 studies resulted in an overall posterior sensitivity of 0.804 (95% credible interval (CI) 0.225–1.0, **Fig 2**), the specificity was 0.853 (95% 0.278–1.00). When accounting for the imperfect nature of the reference test the pooled sensitivity was 0.820 (95% CI 0.15–1.00), the specificity was 0.939 (0.301–1.00)

### Influence of the reference standard test on the diagnostic accuracy of BS

**Fig 3:**
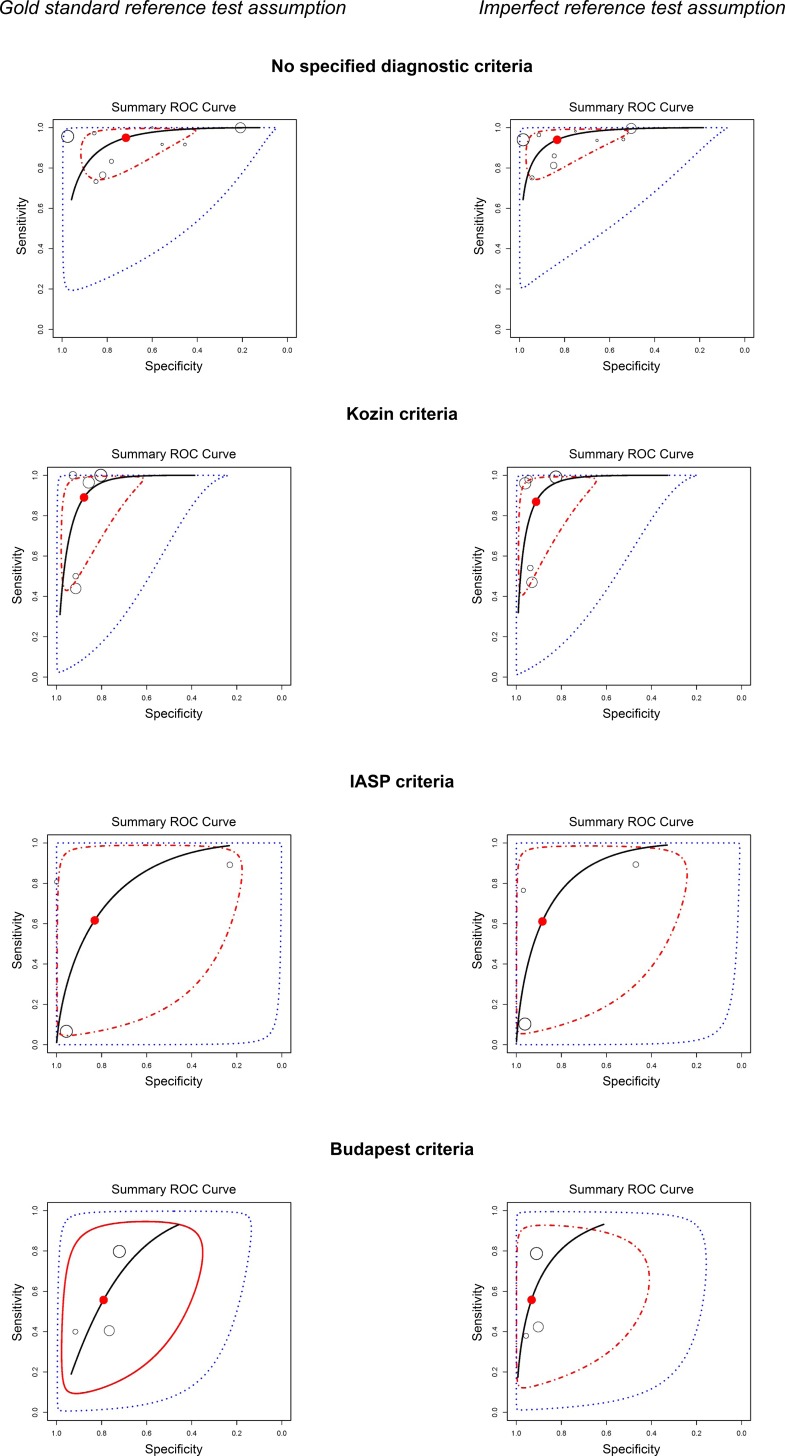
Summary receiver operating characteristic (ROC) curves for different reference standard test. Results of the joint meta-analysis are presented by the overall posterior sensitivity and specificity with the corresponding 95% credible region (CI) IASP, the International Association for the study of pain (IASP).

The joint meta-analysis (Table 2) of studies without diagnostic criteria for CRPS resulted in a posterior sensitivity of 0.933 (95% credible interval (CI) 0.397–1). **Fig 3**visualizes the impact of the different diagnostic reference tests on the summary ROC curve. The joint meta-analysis of studies using Kozin criteria resulted in a posterior sensitivity was 0.814 (95% CI 0.173–1). The posterior sensitivity in studies using the IASP criteria was 0.611 (95% CI 0.005–1) and in Budapest criteria 0.543 (95% CI 0.046–1). When the imperfect reference standard tests were accounted for by the model, the posterior sensitivity further decreased.

**Table 2:** Meta-analysis of diagnostic accuracy for bone scintigraphy.

	Sensitivity perfect reference Standard (95% CI)	Sensitivity imperfect Reference Standard (95% CI)	Specificity perfect reference standard (95% CI)	Specificity imperfect reference standard (95% CI)
No diagnostic criteria	0.933 (0.397; 1)	0.933 (0.395; 1)	0.72 (0.122; 1)	0.829 (0.204; 1)
Kozin criteria	0.814 (0.173;1)	0.806 (0.139; 1)	0.931 (0.325; 1)	0.946 (0.344; 1)
IASP	0.611 (0.005; 1)	0.608 (0.008; 1)	0.830 (0.026; 1)	0.897 (0.045; 1)
Budapest criteria	0.543 (0.046; 1)	0.551 (0.046; 1)	0.89 (0.175; 1)	0.935 (0.306; 1)

95% CI, 95% credible region resulting from the Bayesian analysis calculated from the posterior means (50% quantile), lower (2.5% quantile) and upper (97.5% quantile) bounds.

Perfect reference test, the model assumption is that the bone scintigraphy is compared to a perfect diagnostic reference standard.

Imperfect reference test, the model assumption is that the bone scintigraphy is compared to an imperfect diagnostic reference standard.

The posterior specificity increased from 0.72 (95 CI 0.122–1) in studies that used no diagnostic criteria to 0.935 (95% CI 0.306–1) when Budapest criteria were used and we accounted for the imperfect nature of the reference standard.

### Factors associated with a positive bone scintigraphy

For the analysis of factors associated with positive BS 13 studies [29, 34, 42, 43, 45, 47, 50–56] were analyzed: 7 studies using IASP or Budapest clinical criteria as reference standard reported sensitivity and specificity and 6 studies reported BS results in patients that fulfilled the diagnostic criteria (IASP or Budapest clinical criteria) for the diagnosis of CRPS 1 (Table 1). Longer disease duration showed a negative correlation with positive BS (r = -0.4, p = 0.02, Table 3). Disease duration of less than 52 weeks was associated with more positive BS scans (Wilcoxon rank sum test p = 0.047). Further we found a decreased likelihood for a positive BS with an increasing proportion of women in the study population (r = -0.63, p = 0.009). Age, study design (prospective, retrospective), location of CRPS (upper extremity vs. mixed), were not associated with the sensitivity of the BS.

**Table 3:** Factors associated with positive bone scintigraphy.

Factor	Direction of association	p-value	Statistical test
Duration (dichotomized)	Mean S* ≤ 52 weeks = 0.72	0.047	Wilcoxon rank sum
	Mean S > 52 weeks = 0.58		
Mean duration (continuous)	r** = - 0.4	0.020	Spearman
Mean age	r** = 0.18	0.331	Spearman
Percentage of women	r** = - 0.63	0.009	Spearman
Study design	Mean S prospective = 0.65	0.777	Wilcoxon rank sum
	Mean S retrospective = 0.72		
Location	Mean S UE*** group = 0.67	0.724	Wilcoxon rank sum
	Mean S mixed**** group = 0.73		

* S = sensitivity

**Spearman correlation coefficient

*** UE = upper extremities

**** mixed = UE and lower extremities

## Discussion

The main results of this Bayesian meta-analysis of 21 studies on the test accuracy of bone scintigraphy were twofold. First, Bayesian meta-analysis of studies using the Budapest criteria, recommended since 2007 for the diagnosis of CRPS 1, resulted in a low posterior sensitivity (0.54, 95% credible interval (CI) 0.05–1) and a high posterior specificity (0.89 95% CI 0.18–1). When accounting for the imperfect nature of the reference standard, the sensitivity decreased and the specificity increased (posterior sensitivity 0.55, 95% CI 0.05–1, posterior specificity of 0.94, 95% CI 0.31–1). In studies that did not use a reference standard, sensitivity was high and specificity was low.

Second, disease duration of less than 12 months was associated with higher proportion of positive bone scans compared to disease duration of more than 12 months. Further, a higher proportion of males were associated with more positive scans. How this translates into the diagnostic accuracy of BS in early disease and whether a positive BS in patients with CRPS 1 may be a prognostic factor is unclear.

### Results compared to the literature

The Budapest diagnostic criteria are established for the diagnosis of CRPS 1. The clinical usefulness of BS remains controversial. While some studies support the use of BS for establishing the diagnosis of CRPS [29, 34, 42] others reported a low diagnostic value of a positive BS.[33, 44] In a recent review [57] the authors stated that little emphasis is given to the typical imaging and advocated for the use of BS to support the clinical diagnosis. In a meta-analysis BS was compared to MRI for the diagnosis of CRPS 1 and found a higher sensitivity of BS compared to MRI and a comparable specificity.[58] The authors concluded that BS is more helpful to rule out CRPS 1 than MRI. Our study is the first that used Bayesian meta-analysis methods that account for the prevalence of the disease. Further, we accounted for the imperfect nature of the different reference standard tests. We demonstrated that the high sensitivity reported in many previous studies is mainly due to a lack of the use of a reference standard test. The current study highlights the relevance of the independent nature of the diagnostic test under investigation and the reference test for the diagnosis of the disease.[13] The meta-analysis by Ringer et al. [9] reported results in addition to the summary estimate for all diagnostic studies also the results for the subgroup of studies that used clinical diagnostic criteria and found a higher Sensitivity (0.80, 95% confidence interval 0.44 to 0.95) and a lower specificity 0.73 (0.40 to 0.91).[9] We included three additional studies [43–45] with IASP or more recent clinical diagnostic criteria as reference test. Further, the authors did not account for additional factors that may influence the outcome of diagnostic studies. In addition to the absence of a perfect reference test, participating physicians might have been aware of the results of BS when establishing the reference diagnosis, and therefore, conditional dependence need to be expected. Bayesian meta-analytical methods allow to account for a conditional dependence and also to include covariance terms such as clinical factors that may influence the likelihood of a positive test results (e.g. disease duration, sex, clinical presentation).[10] By accounting for the imperfect nature of the reference test, the conditional dependence, and covariates, we previously described that the heterogeneity between diagnostic studies could be reduced and a better model fit achieved.[10] Despite these methodological advantages of the Bayesian approach, our study also demonstrates the impact of the differences in sensitivity and specificity of the clinical diagnostic criteria (reference tests) on the posterior sensitivity and specificity. Previous external validation study showed for the IASP clinical criteria a high sensitivity(0.98) and a poor specificity (0.36) [19]. The Budapest clinical criteria retained the high sensitivity (0.99), but showed an improved specificity (0.68) [24].

Our findings support clinical guidelines which do not recommend the use of BS for the confirmation of the diagnosis of CRPS 1.[25, 26, 59, 60] While our analysis demonstrated that shorter disease duration is associated with a higher likelihood of a positive BS, it is unclear how this can be used in clinical practice. It may be hypothesized that the higher rate of positive bone scans within the first year is related to the neurogenic inflammation which also may affect bone metabolism.[61] To date, insufficient studies are available to evaluate the prognostic relevance of a positive BS in patients with early CRPS 1 [61]. Despite the fact that women are up to four times more likely to be affected by CRPS 1, we found that men were more likely to have a positive scan. The mechanism explaining this finding is unclear and warrants further investigation.

### Strengths and limitations

This review comprehensively evaluates the currently available studies and this is the first study that uses Bayesian meta-analysis methods to assess the diagnostic accuracy of BS. The search was inclusive, no language restrictions were applied, and a thorough bibliographic search was conducted to identify all relevant studies. The data extraction process was performed in accordance with current guidelines and supported by an experienced statistician. Potential factors influencing diagnostic test accuracy were identified by a multidisciplinary team (an internist, specialist in physical medicine and rehabilitation, statistician, and methodologist).

The study was limited by the small number of studies using a reference test for the diagnosis of CRPS 1. Furthermore, many studies were only of moderate or low quality and some of small sample size. Small studies on diagnostic accuracy are often imprecise, with wide confidence intervals. The lack of a gold standard reference test is another limitation, which we addressed within the Bayesian model formulation; however, the resulting posterior credible intervals for overall sensitivity and specificity of the index test are wider than they would be with a perfect reference test. Only few studies reported factors that influence sensitivity and therefore, the findings need to be interpreted with caution and addressed in future studies.

### Implications for research

Future research should study whether positive BS in CRPS patients is a prognostic factor of the disease. Several treatment strategies include pharmaceuticals that act in the bone metabolism, including bisphosphonates and calcitonin.[62] It may be hypothesized that patients with positive BS respond better to pharmaceutical treatments that influence the bone turn-over, compared to patients with negative scans and therefore, represent a subgroup of CRPS 1 patients.

### Implications for clinical practice

Based on the results of our study BS does not add any value to the clinical diagnosis of CRPS 1 and cannot be used to confirm the diagnosis. Clinicians need to be aware of this fact when communication a positive BS scans to their patients. The diagnosis of CRPS is based on signs and symptoms according to the current diagnostic criteria.[2] Positive BS scans without the corresponding clinical signs and symptoms may result in substantial distress for patients. A negative BS may help to exclude the disease or to rule out other underlying diseases.

### Conclusion

Compared to the accepted Budapest diagnostic criteria BS cannot be used to rule-in the diagnosis of CRPS 1. In patients with negative BS CRPS 1 is less likely the underlying illness. Studies using older or no diagnostic criteria should not be used to evaluate the diagnostic accuracy of BS in CRPS 1.

## Supporting information

S1 Table Prisma 2009 Checklist. (DOC)

S2 Table Details database search. (DOCX)

S3 Table Diagnostic criteria of CRPS 1. (DOCX)

S4 Table Overview of the extracted study results of all studies. (DOCX)

S5 Table Study quality [16]. (DOCX)
