# Supplementary material for: Usefulness of bone scintigraphy for the diagnosis of Complex Regional Pain Syndrome 1: A systematic review and Bayesian meta-analysis
Source: PLoS One. 2017 Mar 16;12(3):e0173688. doi: 10.1371/journal.pone.0173688 (PMC5354289; doi:10.1371/journal.pone.0173688)
Supplement: S2 Table — (DOCX) [file pone.0173688.s002.docx]

**S2 Table. Details database search**

Embase

| # | Query | Results |
| --- | --- | --- |
| 1 | 'complex regional pain syndrome'/exp OR 'complex regional pain syndrome' OR 'complex regional pain syndrome type i'/exp OR 'complex regional pain syndrome type i' | 8,505 |
| 2 | crps:ab,ti OR complex:ab,ti AND regional:ab,ti AND pain:ab,ti AND syndrome*:ab,ti OR rnd:ab,ti OR crps1:ab,ti | 3,679 |
| 3 | posttrauma*:ab,ti AND dystroph*:ab,ti OR (post:ab,ti AND trauma*:ab,ti AND dystroph*:ab,ti) OR (reflex*:ab,ti AND neurovascular:ab,ti AND dystroph*:ab,ti) | 393 |
| 4 | reflex*:ab,ti AND sympathetic:ab,ti AND dystroph*:ab,ti OR (sudeck*:ab,ti AND atroph*:ab,ti) OR algodystroph*:ab,ti OR algoneurodystroph*:ab,ti | 3,067 |
| 5 | (algo NEAR/3 (dystroph* OR neurodystroph*)):ab,ti | 45 |
| 6 | (('shoulder hand' OR 'shoulder hand') NEAR/3 (syndrom* OR dystroph*)):ab,ti | 376 |
| 7 | cervical AND sympathetic AND dystroph*:ab,ti | 95 |
| 8 | #1 OR #2 OR #3 OR #4 OR #5 OR #6 OR #7 | 10,309 |
| 9 | 'bone scintiscanning'/exp | 22,912 |
| 10 | ((bone OR skeleton OR skeletal) NEAR/3 (scan OR scanning OR scintigram OR scintigraphy OR scintiscanning OR scintimetry)):ab,ti | 16,091 |
| 11 | osteoscanning:ab,ti OR osteoscintigraphy:ab,ti OR osteoscintiscanning:ab,ti OR osteoscintimetry:ab,ti OR osteoscintigram:ab,ti | 42 |
| 12 | #9 OR #10 OR #11 | 28,700 |
| 13 | #8 AND #12 | 563 |
| 14 | juvenile'/exp NOT ('juvenile'/exp AND 'adult'/exp) | 1,894,713 |
| 15 | #13 NOT #14 | 525 |

Search Embase, Reed Elsevier Properties SA, July 31, 2015

Medline

| # | Query | Results |
| --- | --- | --- |
| 1 | complex regional pain syndromes/ or reflex sympathetic dystrophy/ | 4115 |
| 2 | (CRPS or complex regional pain syndrome$ or RND or CRPS1).ti,ab. | 2630 |
| 3 | ((posttrauma$ or (post adj3 trauma$) or (reflex$ adj3 neurovascular)) adj3 dystroph$).ti,ab. | 133 |
| 4 | ((reflex$ adj3 sympathetic adj3 dystroph$) or (sudeck$ adj3 atroph$) or algodystroph$ or algoneurodystroph$).ti,ab. | 2204 |
| 5 | (algo adj3 (dystroph$ or neurodystroph$)).ti,ab. | 16 |
| 6 | (("shoulder hand" or "shoulder-hand") adj3 (syndrom$ or dystroph$)).ti,ab. | 288 |
| 7 | (cervical adj3 sympathetic adj3 dystroph$).ti,ab. | 2 |
| 8 | 1 or 2 or 3 or 4 or 5 or 6 or 7 | 6111 |
| 9 | exp "Bone and Bones"/ri [Radionuclide Imaging] | 7746 |
| 10 | ((bone or skeleton or skeletal) adj3 (scan or scanning or scintigram or scintigraphy or scintiscanning or scintimetry)).ti,ab. | 10896 |
| 11 | (osteoscanning or osteoscintigraphy or osteoscintiscanning or osteoscintimetry or osteoscintigram).ti,ab. | 35 |
| 12 | 9 or 10 or 11 | 15403 |
| 13 | 8 and 12 | 256 |
| 14 | animals/ not (animals/ and humans/) | 3828812 |
| 15 | 13 not 14 | 253 |
| 16 | limit 15 to "all child (0 to 18 years)" | 53 |
| 17 | limit 16 to "all adult (19 plus years)" | 31 |
| 18 | 16 not 17 | 22 |
| 19 | 15 not 18 | 231 |

Present Search Strategy: 31.07.2015

Ovid MEDLINE(R) In-Process & Other Non-Indexed Citations, Ovid MEDLINE(R) Daily, Ovid MEDLINE(R) and Ovid OLDMEDLINE(R) 1946 to present

Scopus

( ( TITLE-ABS-KEY ( complex W/3 regional W/3 pain W/3 syndrome* ) ) OR ( TITLE-ABS-KEY ( ( crps OR rnd OR crps1 ) ) ) OR ( TITLE-ABS-KEY ( ( posttrauma* OR ( post W/3 trauma* ) OR ( reflex* W/3 neurovascular ) ) W/3 dystroph* ) ) OR ( TITLE-ABS-KEY ( ( reflex* W/3 sympathetic W/3 dystroph* ) OR ( sudeck* W/3 atroph* ) OR algodystroph* OR algoneurodystroph* ) ) OR ( TITLE-ABS-KEY ( algo W/3 ( dystroph* OR neurodystroph* ) ) ) OR ( TITLE-ABS-KEY ( ( "shoulder hand" OR "shoulder-hand" ) W/3 ( syndrom* OR dystroph* ) ) ) OR ( TITLE-ABS-KEY ( cervical W/3 sympathetic W/3 dystroph* ) ) ) AND ( ( TITLE-ABS-KEY ( ( bone OR skeleton OR skeletal ) W/3 ( scan OR scanning OR scintigram OR scintigraphy OR scintiscanning OR scintimetry ) ) ) OR ( TITLE-ABS-KEY ( osteoscanning OR osteoscintigraphy OR osteoscintiscanning OR osteoscintimetry OR osteoscintigram ) ) )

Present Search Strategy: 31.07.2015 Results: 545
