# Supplementary material for: Usefulness of bone scintigraphy for the diagnosis of Complex Regional Pain Syndrome 1: A systematic review and Bayesian meta-analysis
Source: PLoS One. 2017 Mar 16;12(3):e0173688. doi: 10.1371/journal.pone.0173688 (PMC5354289; doi:10.1371/journal.pone.0173688)
Supplement: S3 Table — (DOCX) [file pone.0173688.s003.docx]

**S3 Table. Diagnostic criteria of CRPS 1**

| **Budapest clinical diagnostic criteria, 2007 [2]** | **Bruehl, 1999 [19]** | **IASP-Orlando, 1994 [20]** | **Veldman, 1993 [21]** | **Kozin, 1981 [22]** | **Steinbrocker, 1958 [23]** |
| --- | --- | --- | --- | --- | --- |
| 1. Continuing pain, which is disproportionate to any inciting event | 1. Continuing pain which is disproportionate to any inciting event | 1. Develops after an initiating noxious event (type I) or after a nerve injury (type II) | 1. 4 or 5 of: • Unexplained diffuse pain • Difference in skin color relative to other limb • Diffuse edema • Difference in skin temperature relative to other limb • Limited active range of motion | Definite: • Pain and tenderness in the distal extremity • Signs and/or symptoms of vasomotor instability • Swelling in the extremity – often with periarticular prominence (dystrophic skin changes usually present) | Stage 1 ('acute'):  • severe pain, burning or aching quality, increased by dependency of the affected part, physical contact or emotional upset.  • Edema  • Hyperthermia or hypothermia  • Increased hair and nail growth occur in the affected past.  • Bony changes may be present on roentgenograms. |
| 2. Must report at least one symptom in three of the four following categories: • Sensory: reports of hyperesthesia and/or allodynia • Vasomotor: reports of temperature asymmetry and/or skin color changes and/or skin color asymmetry • Sudomotor/edema: reports of edema and/or sweating changes and/or sweating asymmetry • Motor/trophic: reports of decreased range of motion and/or motor dysfunction (weakness, tremor, dystonia) and/or trophic changes (hair, nail, skin) | 2. Must report at least one symptom in each of four following categories • Sensory: reports of hyperesthesia • Vasomotor: reports of temperature asymmetry and/or skin color change and/or skin color asymmetry • Sudomotor/edema: reports of edema and/or sweating changes and/or sweating asymmetry • Motor/trophic: reports of decreased range of motion and/or motor dysfunction (weakness, tremor, dystonia) and/or trophic changes (hair, nail, skin) | 2. Spontaneous pain or allodynia/hyperalgesia that is not limited to the territory of a single peripheral nerve and is disproportionate to the inciting event | 2. Occurrence or increase of above signs and symptom after use | Probable: • Pain and tenderness and • Vasomotor instability or swelling (dystrophic skin changes often present) | Stage 2 ('dystrophic' stage):  • Dystrophic changes and the persistence of pain and disability. The edematous tissue becomes indurated and the skin is cool and hyperhidrotic.  • Roentgenogram may reveal diffuse osteoporosis. |
| 3. Must display at least one sign at time of evaluation in two or more of the following categories: • Sensory: evidence of hyperalgesia (to pinprick) and/or allodynia (to light touch and/or deep somatic pressure and/or joint movement) • Vasomotor: evidence of temperature asymmetry and/or skin color changes and/or asymmetry • Sudomotor/edema: evidence of edema and/or sweating changes and/or sweating asymmetry • Motor/trophic: evidence of decreased range of motion and/or motor dysfunction (weakness, tremor, dystonia) and/or trophic changes (hair, nail, skin) | 3. Must display at least one sign in two or more of the following categories: • Sensory: evidence of hyperalgesia (to pinprick) and/or allodynia (to light touch) • Vasomotor: evidence of temperature asymmetry and/or skin colour changes and/or asymmetry • Sudomotor/oedema: evidence of oedema and/or sweating changes and/or sweating asymmetry • Motor/trophic: evidence of decreased range of motion and/or motor dysfunction (weakness, tremor, dystonia) and/or trophic changes (hair, nail, skin) | 3. There is or has been evidence of oedema, skin blood flow abnormality, or abnormal sudomotor activity in the region of the pain since the inciting event | 3. Above signs and symptoms present in an area larger than the area of primary injury or operation and including the area distal to the primary injury | Possible • Vasomotor instability and/or swelling • No pain, but mild moderate tenderness may be present (dystrophic skin changes occasionally present) | Stage 3 ('atrophic' stage):  • Progressive skin and subcutaneous tissue atrophy, and occasionally proximal spread of pain. The skin is thin and shiny, the fascia becomes thickened and flexion or Dupuytren's contractures may occur.  • Roentgenogsams show masked demineralisation and ankylosis. |
| 4. There is no other diagnosis that better explains the signs and symptoms |  | 4. There is no other diagnosis that better explains the degree of pain and dysfunction  Note: criteria 2-4 must be satisfied |  | Doubtful • Unexplained pain and tenderness in an extremity |  |
