# Supplementary material for: Usefulness of bone scintigraphy for the diagnosis of Complex Regional Pain Syndrome 1: A systematic review and Bayesian meta-analysis
Source: PLoS One. 2017 Mar 16;12(3):e0173688. doi: 10.1371/journal.pone.0173688 (PMC5354289; doi:10.1371/journal.pone.0173688)
Supplement: S4 Table — (DOCX) [file pone.0173688.s004.docx]

**S4 Table.** **Overview of the extracted study results of all studies**

| ID | **Author, year** | **Design** | **Patients** | **W: n (%)** | **Age** | **SD (weeks)** | **Ref. Sd.** | **TP** | **FP** | **FN** | **TN** | **Sens** | **Spec** |
| --- | --- | --- | --- | --- | --- | --- | --- | --- | --- | --- | --- | --- | --- |
| 2 | Kozin, 1981 | P | 50 | 28 (56) | 48.3 | 75.9 | NR | 15 | 7 | 3 | 25 | 0.83 | 0.78 |
| 3 | Leitha, 1996 | P | 120 | 82 (68) | 50.1 | 36.8 | NR | 96 | 19 | 0 | 5 | 1.00 | 0.21 |
| 4 | O'Donoghue, 1993 | P | 78 | N.R. | N.R. | N.R. | NR | 13 | 11 | 4 | 50 | 0.76 | 0.82 |
| 8 | Schiepers, 1998 | R | 50 | 27 (54) | 44 | N.R. | NR | 22 | 3 | 8 | 17 | 0.73 | 0.85 |
| 10 | Todorovic, 1995 | R | 44 | 18 (41) | 51 | 8.8 | NR | 36 | 1 | 1 | 6 | 0.97 | 0.86 |
| 16 | Weiss 1993 | R | 22 | N.R. | N.R. | N.R. | NR | 12 | 4 | 0 | 6 | 1.00 | 0.60 |
| 19 | Mackinnon 1983 | R | 145 | N.R. | 43 | N.R. | NR | 22 | 3 | 1 | 119 | 0.96 | 0.98 |
| 11 | Wang, 1998 | R | 30 | 9 (30) | 63 | 6.1 | Clinical | 11 | 8 | 1 | 10 | 0.92 | 0.56 |
| 5 | Okudan, 2005 | P | 34 | 17 (50) | 61 | 6.5 | Clinical | 11 | 12 | 1 | 10 | 0.92 | 0.45 |
| 13 | Constantinesco 1986 | R | 128 | 61 (48) | 51 | 23 | Kozin | 83 | 6 | 3 | 36 | 0.97 | 0.86 |
| 1 A+B | Tepperman 1984 and Greyson 1984 | R | 85 | 37 (44) | 60 | 9 | Kozin | 16 | 5 | 0 | 64 | 1.00 | 0.93 |
| 15 | Werner 1988 | R | 63 | N.R. | 38 | 84 | Kozin | 8 | 4 | 8 | 43 | 0.50 | 0.91 |
| 17 | Holder 1992 | M | 138 | 18 (13) | 43.5 | 24 | Kozin | 26 | 22 | 0 | 90 | 1.00 | 0.80 |
| 18 | Davidoff 1989 | R | 119 | 65 (55) | 35.1 | 103.6 | Kozin | 11 | 8 | 14 | 86 | 0.44 | 0.91 |
| 9 | Schurmann, 2007 | P | 107 | 75 (70) | 59.9 | 16 | IASP | 1 | 4 | 14 | 88 | 0.07 | 0.96 |
| 21 | Kim 2015 | R | 13 | 5 (38) | 51 | N.R. | IASP | 5 | 1 | 1 | 6 | 0.83 | 0.86 |
| 6 | Park, 2007 | R | 38 | 13 (34) | 52.2 | 0.3 | IASP | 21 | 0 | 5 | 12 | 0.81 | 1.00 |
| 7 | Park, 2009 | P | 50 | 27 (54) | 56 | 13 | IASP | 33 | 10 | 4 | 3 | 0.89 | 0.23 |
| 7 | Park, 2009 | P | 14 | N.R. | 56 | 2-6 | IASP | 1 | 1 | 9 | 3 | 0.10 | 0.75 |
| 7 | Park, 2009 | P | 24 | N.R. | 56 | 7-20 | IASP | 15 | 1 | 3 | 5 | 0.83 | 0.83 |
| 7 | Park, 2009 | P | 9 | N.R. | 56 | 21-28 | IASP | 7 | 3 | 2 | 1 | 0.78 | 0.25 |
| 20 | Kwon 2011 | R | 140 | 60 (43) | 39 | 64 | Budapest | 63 | 17 | 16 | 44 | 0.80 | 0.72 |
| 14 | Moon 2012 | R | 116 | 50 (43) | 40.5 | 53.6 | Budapest | 28 | 11 | 41 | 36 | 0.41 | 0.77 |
| 12 | Wüppenhorst, 2010 | P | 57 | 38 (67) | 50.7 | 56.8 | Bruehl | 18 | 1 | 27 | 11 | 0.40 | 0.92 |
| 44 | Konzelmann | R | 15 | 5 (33) | 43 | 7.8 | Budapest criteria | 12 | N/A | 3 | N/A | 0.80 | N/A |
| 44 | Konzelmann | R | 8 | N.R. | 43 | <6 months | Budapest criteria | 8 | N/A | 0 | N/A | 1.00 | N/A |
| 44 | Konzelmann | R | 7 | N.R. | 43 | >6 months | Budapest criteria | 5 | N/A | 2 | N/A | 0.71 | N/A |
| 22 | AlSharif, 2012 | R | 37 | 16 (43) | 38.8 | 6.3 | IASP | 25 | N/A | 12 | N/A | 0.68 | N/A |
| 22 | AlSharif, 2012 | R | 19 | N.R. | 38.8 | <3months | IASP | 16 | N/A | 3 | N/A | 0.84 | N/A |
| 22 | AlSharif, 2012 | R | 18 | N.R. | 38.8 | >3 months | IASP | 9 | N/A | 9 | N/A | 0.50 | N/A |
| 22 | AlSharif, 2012 | R | 37 | N.R. | 38.8 | Subgroup vasomotor symptoms | IASP | 22 | N/A | 6 | N/A | 0.79 | N/A |
| 22 | AlSharif, 2012 | R | 37 | N.R. | 38.8 | Subgroup motor / trophic changes | IASP | 18 | N/A | 3 | N/A | 0.86 | N/A |
| 40 | Handa, 2006 | R | 14 | 6 (43) | 55 | 3 | IASP | 14 | N/A | 0 | N/A | 1.00 | N/A |
| 53 | Sampath, 2013 | R | 68 | 38 (56) | 43 | N.R. | IASP | 37 | N/A | 31 | N/A | 0.54 | N/A |
| 53 | Sampath, 2013 | R | 22 | N.R | 43 | 0-20weeks | IASP | 15 | N/A | 7 | N/A | 0.68 | N/A |
| 53 | Sampath, 2013 | R | 18 | N.R | 43 | 20-60 weeks | IASP | 14 | N/A | 4 | N/A | 0.78 | N/A |
| 53 | Sampath, 2013 | R | 12 | N.R | 43 | >60 weeks | IASP | 8 | N/A | 4 | N/A | 0.67 | N/A |
| 55 | Sezer, 2008 | P | 24 | 19 (79) | 52 | 1.4 | IASP | 23 | N/A | 1 | N/A | 0.96 | N/A |
| 55 | Sezer, 2008 | P | 13 | N.R. | 52 | ≤1 months | IASP | 12 | N/A | 1 | N/A | 0.92 | N/A |
| 55 | Sezer, 2008 | P | 11 | N.R. | 52 | >1-3months | IASP | 11 | N/A | 1 | N/A | 0.92 | N/A |
| 26 | Bruehl, 2002 | R | 38 | 24 (63) | 41 | 26.9 | IASP | 12 | N/A | 26 | N/A | 0.32 | N/A |
| 26 | Bruehl, 2002 | R | 12 | N.R. | 43.6 | Cluster 1, vasomotor dysfunction, SD 27months | IASP | 11 | N/A | 1 | N/A | 0.92 | N/A |
| 26 | Bruehl, 2002 | R | 9 | N.R. | 40.4 | Cluster 2, pain and sensory, SD 34.6 months | IASP | 6 | N/A | 3 | N/A | 0.67 | N/A |
| 26 | Bruehl, 2002 | R | 17 | N.R. | 39.8 | Cluster 3: florid with high vasomotor, pain symptoms, SDD 23.3 months | IASP | 9 | N/A | 8 | N/A | 0.53 | N/A |

Abbreviations: W, women; SD, symptom duration; Ref. Sd., reference standards; TP, true positive; FP, false positive; FN, false negative; TN, true negative; Sens, sensitivity; Spec, specificity; R, retrospective study design; P, prospective study design; M, mixed retrospective and prospective study design; N.R., not reported; N/A, not applicable
