# Supplementary material for: Usefulness of bone scintigraphy for the diagnosis of Complex Regional Pain Syndrome 1: A systematic review and Bayesian meta-analysis
Source: PLoS One. 2017 Mar 16;12(3):e0173688. doi: 10.1371/journal.pone.0173688 (PMC5354289; doi:10.1371/journal.pone.0173688)
Supplement: S5 Table — (DOCX) [file pone.0173688.s005.docx]

**S5 Table. Study quality [16]**

| **Author, year** | Overall Quality | consecutive sequence or random selection | Case-control methods not used | included patients match the key question | Inappropriate exclusion avoided | Index text interpretation without knowledge of reference standard | Index test threshold pre-specifived | Index test interpretation similar to guidelines | reference standard likely to identify target condition | reference standard interpreted without knowledge of IT | condition found by RT matches guidelines | interval between IT and RT appropriate | same RT in all patients | All patients included analyzed |
| --- | --- | --- | --- | --- | --- | --- | --- | --- | --- | --- | --- | --- | --- | --- |
| Kozin, 1981 | + | Yes | Yes | Yes | Yes | Can't say | Yes | Yes | No | Yes | No | Yes | Yes | No |
| Leitha, 1996 | + | Yes | Yes | Yes | Yes | Can't say | Yes | Can't say | No | Can't say | No | Yes | Yes | Yes |
| O'Donoghue, 1993 | + | Yes | Yes | Can't say | Yes | Yes | Yes | Yes | No | Yes | Yes | Yes | Yes | Yes |
| Okudan, 2005 | + | Yes | Yes | Can't say | Can't say | Can't say | Yes | Yes | Yes | Yes | Yes | Yes | Yes | Yes |
| Park, 2007 | + | Can't say | Yes | Can't say | Yes | Can't say | Yes | Yes | Yes | Yes | Yes | Yes | Yes | Yes |
| Park, 2009 | + | Can't say | Yes | Can't say | Yes | Can't say | Yes | Yes | Yes | Can't say | Yes | Yes | Yes | Can't say |
| Schiepers, 1998 | - | Yes | Yes | Can't say | Yes | Can't say | Yes | Yes | No | Can't say | No | Can't say | Yes | Yes |
| Schurmann, 2007 | + | Yes | Yes | Yes | Yes | Yes | Yes | Yes | Yes | Can't say | Yes | Yes | Yes | Yes |
| Todorovic, 1995 | - | No | Yes | Can't say | Yes | Yes | Yes | No | No | No | No | Yes | Can't say | Yes |
| Wang, 1998 | - | Can't say | Yes | Can't say | Yes | Can't say | Yes | Yes | Yes | Can't say | No | Can't say | Yes | Yes |
| Wüppenhorst, 2010 | ++ | Yes | Yes | Can't say | Yes | Yes | Yes | Yes | Yes | Can't say | Yes | Yes | Yes | Yes |
| Constantinesco 1986 | + | Can't say | Yes | Can't say | Yes | Can't say | Yes | Yes | Yes | Can't say | Yes | Yes | Yes | Yes |
| Moon 2012 | + | Yes | Yes | Yes | Yes | Can't say | Yes | Yes | Yes | Can't say | Yes | Yes | Yes | Yes |
| Tepperman 1984 and Greyson 1984 | + | Can't say | Can't say | Can't say | Yes | Can't say | Yes | Yes | Yes | Can't say | Yes | Yes | Yes | Yes |
| Werner 1988 | + | Can't say | Can't say | Can't say | Yes | Can't say | Yes | Yes | Yes | Can't say | Yes | Yes | Yes | Yes |
| Weiss 1993 | - | Can't say | Yes | Can't say | Yes | Can't say | Yes | Yes | No | Can't say | No | Can't say | Yes | Can't say |
| Holder 1992 | + | Yes | Yes | Can't say | Yes | Can't say | Yes | Yes | Yes | Can't say | Yes | Yes | Yes | Can't say |
| Davidoff 1989 | + | Can't say | Yes | Can't say | Yes | Can't say | Yes | Yes | Yes | Can't say | Yes | Yes | Yes | Yes |
| Mackinnon 1983 | + | Yes | Yes | Can't say | Yes | Can't say | Yes | Yes | No | Can't say | No | Yes | Yes | Yes |
| Kwon 2011 | + | Yes | Yes | Can't say | Yes | Can't say | Yes | Yes | Yes | Can't say | Yes | Yes | Yes | Yes |
| Kim 2015 | - | Can't say | Yes | Can't say | Yes | Can't say | Yes | Yes | Yes | Can't say | Yes | Yes | Yes | Yes |
| Konzelmann | + | No | Yes | Can't say | Yes | No | Yes | Yes | Yes | Can't say | Yes | Yes | Yes | Yes |
| AlSharif, 2012 | + | Can't say | Yes | Yes | Yes | Can't say | Yes | Yes | Yes | Can't say | Yes | Can't say | Yes | Yes |
| Handa, 2006 | + | Can't say | Yes | Can't say | Yes | Can't say | Yes | Yes | Yes | Yes | Yes | Yes | Yes | Yes |
| Sampath, 2013 | + | Can't say | Yes | Can't say | Yes | Can't say | Yes | Yes | Yes | Can't say | Yes | Yes | Yes | Yes |
| Sezer, 2008 | + | Yes | Yes | Yes | Yes | No | Yes | Yes | Yes | Can't say | Yes | Can't say | Yes | Yes |
| Brühl 2002 | - | Yes | Yes | Yes | Yes | Can't say | Can't say | Yes | Yes | No | Can't say | Can't say | Yes | Can't say |

++, high quality; +, moderate quality; -, low quality
